# Venoms of Iranian Scorpions (Arachnida, Scorpiones) and Their Potential for Drug Discovery

**DOI:** 10.3390/molecules24142670

**Published:** 2019-07-23

**Authors:** Seyed Mahdi Kazemi, Jean-Marc Sabatier

**Affiliations:** 1 Zagros Herpetological Institute, No 12, Somayyeh 14 Avenue, 3715688415 Qom, Iran; 2 Institute of NeuroPhysiopathology, UMR 7051, Faculté de Médecine Secteur Nord, 51, Boulevard Pierre Dramard-CS80011, 13344-Marseille Cedex 15, France

**Keywords:** scorpion, fauna, venom, toxin, Iran

## Abstract

Scorpions, a characteristic group of arthropods, are among the earliest diverging arachnids, dating back almost 440 million years. One of the many interesting aspects of scorpions is that they have venom arsenals for capturing prey and defending against predators, which may play a critical role in their evolutionary success. Unfortunately, however, scorpion envenomation represents a serious health problem in several countries, including Iran. Iran is acknowledged as an area with a high richness of scorpion species and families. The diversity of the scorpion fauna in Iran is the subject of this review, in which we report a total of 78 species and subspecies in 19 genera and four families. We also list some of the toxins or genes studied from five species, including *Androctonus crassicauda*, *Hottentotta zagrosensis, Mesobuthus phillipsi, Odontobuthus doriae,* and *Hemiscorpius lepturus*, in the Buthidae and Hemiscorpiidae families. Lastly, we review the diverse functions of typical toxins from the Iranian scorpion species, including their medical applications.

## 1. Introduction

Scorpions, a characteristic group of arthropods, diverged from other arachnids relatively early, at about 440 million years ago [1,2]. Their morphology has stayed constant since they adapted to a terrestrial habitat. Their morphological stasis has not been, however, an impediment to successfully colonizing different ecological ecosystems such as caves, high peaks, and deserts [3,4].

Morphological phylogenetic analyses suggest that scorpions are sister taxa to either the rest of the arachnids or to the Opiliones [5]. However, recent phylogenomic analysis suggests that scorpions are closely related to spiders and allies, forming the clade Arachnopulmonata [6,7].

Among the 18 scorpion families with more than 2200 species described in the world, about thirty species have been identified as potentially deadly toxic to humans [8,9].

Our studies on scorpions are based on two remarkable aspects of this group: Their evolutionary origin and systematic classification, and the diversity and origin of their venom components, with emphasis on the use of these components as potential sources of molecules with therapeutic applications [10].

Scorpions have fascinated scientists and laypersons for their venom, which is a complex mixture of bioactive components secreted in specialized organs [11]. These animals inject venom to subdue prey or to defend against attackers. Their venoms consist of a variety of toxins, which may vary according to species, habitat, or fluctuations in climate [12]. Within scorpion venom components, the peptidic fraction has been considered a great source of lead compounds for drugs to treat various cancers and infectious diseases [13]. Hence, studies on scorpion venom components are important, especially in terms of medical treatments for human diseases.

Here, we review the diversity of scorpions in Iran. We also highlight the importance of the venomic studies of Iranian scorpions. Establishing what is known about of scorpions and scorpion venom in Iran will allow the identification of important gaps to be addressed in the future.

## 2. Scorpion Species from Iran

Iran is a vast land with diverse climates. There are two main mountain ranges in Iran: (1) Alborz; and (2) Zagros and neighboring mountains. Diverse climates in Iran are the direct result of the presence of the Persian Gulf, the Oman Sea bordering Southeastern Iran, and the Caspian Sea in Northern Iran. According to Safaei-Mahroo et al. [14] 16 terrestrial ecoregions have been reported from Iran including: Arabian Desert and East Saharo-Arabian xeric shrublands (0.1%), Azerbaijan shrub desert and steppe (0.4%), Badkhiz-Karabil semi-desert (0.1%), Caspian lowland desert (0.3%), Central Persian desert basins (34.7%), Kopet Dagh semi-desert (0.4 %), Registan-North Pakistan sandy desert (3%), South Iran Nubo-Sindian desert and semi-desert (17.3%), Mesopotamian shrub desert (0.1%), Tigris-Euphrates alluvial salt marsh (0.4%), Kopet Dagh woodlands and forest steppe (1.6%), Kuh Rud and Eastern Iran Montane woodlands (7.5%), Caspian Hyrcanian mixed forests (3.4%), Zagros Mountains forest steppe (21.8%), Alborz Range forest steppe (4.3%), and Eastern Anatolian montane steppe (4.6%).

Iran is located in a strategic position in the Palearctic region and is a bridge between the Oriental and African zoogeographical regions, suggesting the possibility of endemic arthropod species in this region. Among these arthropods, scorpions stand out as there are diverse species in desert and semi-desert regions of Iran (for more details see appendix: Table S1).

Historically, the fauna of Iran has been studied by many researchers. The earliest descriptions of the first species were made by Olivier (*Androctonus crassicauda* (Olivier, 1807)) [15]. Later, Alexei Andreevich Byalynitskii-Birulya [16,17,18,19] published a series of scorpion studies from Iran that included well known species and several rare taxa [20,21,22,23,24,25,26]. In addition, pioneering zoologists such as Pocock [27] and Werner [28] described a few more species in the region. In the middle of the 20^th^ century, Max Vachon carried out preliminary studies on the scorpions of Iran, with a later report of two families, nine genera, and 15 species [29]. Later, Habibi [30] reported 24 species belonging to 11 genera and two families. Farzanpay [31,32] reported fewer species (23 species), but increased the number of genera (17), as well as two families. More recently, Kovařík [23] reported a list of three families, 17 genera, and 32 species of scorpions. A more extensive study of Iranian scorpions continued with publications by Navidpour et al. [33,34,35,36,37], who recorded the dispersal of scorpions in all of Iran. Lastly, Mirshamsi et al. [38] reported 51 species belonging to 18 genera in four families. According to Vachon [29] and Mirshamsi et al. [38], there are *Androctonus baluchicus, Androctonus crassicauda,* and *Androctonus finitimus* in Iran. However, Yağmur et al. [39] believed that *Androctonus crassicauda* and *Androctonus robustus* are present in Iran while rejecting the occurrence of *Androctonus baluchicus* and *Androctonus finitimus* in Iran. *Compsobuthus kafkai* and *Compsobuthus sobotniki* were synonymized with *Sassanidotus gracilis* [37,40]. Farzanpay [32] believed that *Hottentotta alticola alticola* are present in Iran but Mirshamsi et al. [38] believed that records show *Hottentotta alticola alticola* are in doubt. Mirshamsi [41] believed that *Mesobuthus phillipsi* includes the *Mesobuthus phillipsi pachysoma* and *Mesobuthus phillipsi mesopotamicus* subspecies. Although Vachon [29], Farzanpay [32], Mirshamsi et al. [38], and Nejati et al. [42], reported *Odontobuthus odonturus* in Iran, Lowe [43] rejected *Odontobuthus odonturus* in Iran. Based on field work, study collections, literature reviews, and personal communications, the total number of species confirmed within the Iranian border is 78 species and subspecies belonging to 19 genera and four families. The family Buthidae is the most diverse with 68 species and subspecies (87.17%), followed by Hemiscorpiidae with seven species (8.97%), Scorpionidae with two subspecies (2.56%), and Diplocentridae with one species (1.28%) [23,24,25,26,29,32,33,34,35,36,37,38,39,40,41,42,44,45,46,47,48,49] (Table S2). Forty-five out of 78 species and subspecies of the Iranian scorpions are endemic to Iran (57.69%, for more details see appendix: Tables S1 and S2).

## 3. Previous Studies on Drug Discovery of Scorpion Venoms

Animal venoms are a mixture of different compounds for defense and prey capture. Many peptide toxins from deadly animal venoms have been influenced by ion channel (including sodium, potassium, and calcium channels) functions. The ion channels play important roles in the regulation of the heart beat and neuronal excitability [50,51].

Scorpion venoms are certainly important natural drug resources for medical applications. In scorpions, family Buthidae has always been interesting from the public health perspective in terms of their dangerous venoms. Many studies have concentrated on non-Buthidae families and reported several new venom peptides and proteins which have shown unique primary structures and biological activities [52,53,54,55]. However, the first disulfide-bridged peptide toxin extracted from a non-buthid scorpion was St20 from *Scorpiops tibetanus*. This peptide has immunosuppressive and anti-inflammatory effects that suggest its potential use as a new peptide medicine for human diseases [56].

Scorpion toxins have been used in variety of fields, including biotechnology (examining the effects on ion channels), identifying cancer mass [57], treating cancer [58], and to treat neuronal [59], autoimmune [60], and cardiovascular diseases [61].

The venoms of *Pandinus imperator* and *Scorpio maurus palmatus* have peptides named imperatoxin A (IpTxa) and maurocalcin (MCa), respectively, and these venoms are of interest in many cardiovascular diseases [62,63].

Classification of polypeptide toxins is important for understanding the structure–function relationship of each individual group. The major criteria used for classification are based on receptor/ion channel specificity (e.g., K^+^, Na^+^, Ca^2+^ and Cl^_^), peptide length (e.g., short- and long-chain), structural scaffold α, αβ and βαββ), disulfide bonds (three or four and pairing pattern), the mechanism of action/binding sites (α- or β-like toxins), their cellular target, and others. [64,65,66]. Ion channels play critical roles in the secretion of hormones, cell proliferation and motility, muscle activity, sense perception, and brain activities of which the functions are applicable for drug development [67,68].

## 4. Venomic Studies in Iranian Scorpions and Their Potential in Therapeutic

Biologically, venoms of scorpions are diverse and have activity due to their predatory and defensive use in nature [69,70]. In addition, venom of scorpions contains phospholipases A_2_, serine proteases, metalloproteases, lipolysis activating peptides (LVPs) and hyaluronidases, proteins, and peptides (antimicrobial and toxic peptides performing on ion channels) [71,72,73].

Several peptide toxins in venomous animals are being considered for pharmacological applications, including treating pain, diabetes, multiple sclerosis, and cardiovascular illnesses [12,51,74].

Worldwide, peptides are progressively emerging as a novel class of therapeutics. In total, 438 peptides are represented in the pharmaceutical trade, including 72 in Phase III clinical trials and 48 that have been approved. Four are famous and sold in the pharmaceutical market: Copaxane, Lupron, Byetta, and Forteo [75]. The majority of these peptides act through G protein-coupled receptors or ion channels [74].

## 5. Scorpion Venom and Cancer Therapy

Several studies have reported that scorpion peptides have antineoplastic activity [76,77]. Some researchers showed that scorpion venoms have potential as a source of drug-like molecules to treat diverse cancers such as human neuroblastoma, leukemia, glioma, brain tumor, breast cancer, melanoma, prostate cancer, and human lung adenocarcinomas [78].

Anti-proliferative, cytotoxic, and apoptogenic properties of scorpion venom peptides on different types of cancers have also been discovered. Scorpion venom peptides with fluorescent labeling have been used to visualize the boundaries of cancerous tissues in cancer patients [78].

For example, the venom of *Androctonus crassicauda* (100 μg/mL) blocked propagation of MCF-7 cells by suppressing S-phase of the cell cycle [79,80,81]. High doses produce necrosis, killing the cells, while apoptosis diminishes cell growth at lower doses, producing inhibition of growth of breast cancer cells [79].

The venom of *Odontobuthus doriae* causes apoptosis in breast cancer by depolarizing mitochondria membranes and controlling S-phase proliferation in human breast cancer cells. MCF-7, on the other hand, reduced catalase activity, glutathione production, DNA fragmentation, and apoptosis [80].

## 6. Scorpion Toxin and Ion Channels

Ion channels include voltage-gated sodium, calcium, and potassium channels which create electrical signals required for action potential generation and conduction, and are the molecular targets for a broad range of potent neurotoxins [82].

### 6.1. Nav or Gated Sodium Channel Specific Toxins

Voltage-gated sodium channels (Na_vs_) are composed of transmembrane proteins that conduct sodium ion (Na^+^) into the cytosol upon activation. An Na_v_ contains a main α-subunit (220–260 kDa) and one or two auxiliary β-subunits (30–40 kDa) [83,84]. The Nav α-subunit is composed of four homologous domains (DI-DIV), each containing six transmembrane α-helixes (S1–S6). The α-subunit includes multiple domains, which are involved in pore-forming, voltage-sensing, and Na^+^ selectivity [85]. Na_vs_ play an important role for action potential (AP) generation and proliferation in excitable cells, including cardiac myocytes, skeletal muscle cells, and neurons [86,87]. Na_vs_ are also marginally expressed in non-excitable cells involved in noncanonical roles in controlling some pathophysiological activities [87,88].

Many studies have focused on roles of Na_v_ subtypes (Na_v_1.3, 1.7, 1.8, and 1.9) in nociceptive transduction. These Na_vs_ probably show attractive targets for analgesic drug discovery. However, their channels also introduce valuable probes to demonstrate the structures, gating properties, and cellular functions of ion channels (for more details see Wu et al. [89].

Three categories of Na_v_ toxins can be defined: (1) Pore-blocking toxins that inhibit Na^+^ conductance by interacting with neurotoxin site 1, including tetrodotoxin (TTX), μ-conotoxins, and saxitoxin (STX); (2) toxins that negatively shift the activation voltage and produce a persistent activation by connecting to membrane-embedded neurotoxin site 2 (such as veratridine, grayanotoxin, and batrachotoxin), or 5 (such as ciguatoxin and brevetoxin), and that prefer to interact with the open state of channel; and (3) toxins that delay inactivation by binding to extracellular neurotoxin site 3, such as sea-anemone toxins and α-scorpion toxins. According to recent investigations, β-scorpion toxins shift activation voltage to either a depolarized or hyperpolarized direction through main effects of neurotoxin site 4. Delta-conotoxin prolongs channel inactivation, similarly to that caused by α-scorpion toxins, by binding to neurotoxin site 6. Although the neurotoxin binding sites are topologically distinct, allosteric coupling has been elucidated between sites 3 and 6 and between sites 2 and 5 [90,91]. In addition, four β-subunits (β1–β4; encoded by SCN*1B*-*4B* genes) have been reported in mammals. The β-subunits are type I transmembrane proteins, including an extracellular signal peptide in the N-terminus, a transmembrane segment, and an immunoglobulin domain [92,93]. Both excitable and non-excitable cells have considerable amounts of β-subunit expression that plays critical roles in modulating the localization, kinetics, and gating of Na_v_ α-subunits [87,91,93,94].

There are several studies that provide evidence of these mechanisms. As described above, there are several kinds of toxins in scorpion venoms that modulate the activity of ion channels, and these are usually responsible for several signs of envenoming. From the human perspective, the most medically important toxins are those that modulate mammalian Na_V_ channels [64,69].

The first neurotoxin targeting voltage-gated sodium channels extracted from an Iranian scorpion venom (*Odontobuthus doriae*) was OD_1_, which blocks the fast inactivation of mammalian channels Na_v_1.7, Na_v_1.4, and Na_v_1.6 [95,96]. Additionally, OD_1_ inhibits fast inactivation of the para/tipE insect channel (EC_50_ 80 nM), but it scarcely influences mammalian Na_v_1.3 or Na_v_1.5 (EC_50_ > 1 μM) and has no effect on Na_v_1.2 and Na_v_1.8. OD_1_ rapidly induces pain when injected into animals in association with, or without, veratridine, and has been used to test the analgesic effects of Na_v_1.7 blockers in vivo [96,97]. In fact, dose-dependent increases in the amplitude of Na^+^ currents resulting in incomplete inactivation in steady-state conditions has been demonstrated by OD_1_ [98,99]. It is probable that continuous inward Na^+^ current causes sustained depolarization of the cell membrane, and the remaining Nav1.7 channels that were not affected by OD_1_ are trapped in the inactivated state, resulting in the loss of electrical excitability of nociceptor neurons [97,98].

*Buthotus schach* venom is an important source of active peptides, some of which affect voltage-gated sodium channels involved in local pain, inflammation, convulsion, necrosis, respiratory depression, and cardiac arrest in humans [100,101].

### 6.2. K^+^ Channel Specific Scorpion Toxins

Potassium channels are the most abundant ion channels and are found in all living organisms [102]. The channels are involved in the resting potential, and shape the action potential, in nerves and muscles [67]. Most potassium channels are composed of tetramers of principal α-subunits (heteromeric assemblies are more common) [103], with auxiliary β-subunits as complementary parts [104]. There are 78 genes encoding α-subunits of potassium channels in the human genome divided into five classes: K_ir_, K_2P_, K_V_, and two groups of K_Ca_ [105,106,107,108].

Based on many studies, potassium channel ligands are probably classified into two large groups. These are pore blockers that physically obstruct the channel pore, and gating modifiers that influence channel traits [82]. The potassium channel ligands can be metal ions, low-molecular-mass substances, and polypeptides [109].

Scorpion venom has K^+^ channel specific toxins (KTx). Thus far, 293 KTxs have been described in UniProt and 174 KTxs have been described in the Kalium database at http://kaliumdb.org/ [110,111]. The toxins interact with different subtypes of channels, such as the K_v1_, K_v3_, K_v4_, K_v7_, K_v11_, and K_Ca_ channels [112].

Venom of scorpions are an important source of K^+^ channel specific toxins (KTx), and are important tools for the structural and functional characterization of various K^+^ [113,114]. K_v_ channel inhibitors have medical applications in the treatment of various specific human diseases, especially autoimmune disorders, inflammatory neuropathies, and cancer [111].

Autoimmune diseases are usually accompanied by tissue injury caused by autoantigen-specific T-cells. K_V_1.3 channels contribute to the control of calcium signaling to induce T-cell proliferation, immune activation, and cytokine production. In many autoimmune diseases, effector memory T (TEM) cells, which play major roles, are controlled by blocking K_V_1.3 channels on the membrane. Animal toxins are capable of suppressing the activation and proliferation of TEM cells and may improve TEM cell-mediated autoimmune diseases, for example in multiple sclerosis and type I diabetes mellitus [115].

The first K^+^ channel toxin isolated from the venom of the Iranian scorpion *Odontobuthus doriae* (OdK_1_) was categorized as α-KTx 8.5. The pharmacological effects of OdK_1_ were investigated on *Xenopus laevis* oocytes heterologously expressing K_v_1.2 channels. OdK_1_ selectively blocked the currents through K_v_1.2 channels, with no effect on the other channels tested [116].

There is a Kv1.3 channel-selective toxin, OdK2, in venom of *Odonthobuthus doriae*, one of the endemic scorpion species of Iran. OdK2 is composed of 38 amino acid residues, including six conserved half-cystine residues and a C-terminal lysine residue. The toxin was named KTX3.11. Pharmacologically, OdK2 selectively suppresses the currents via K_v_1.3 channels. [117].

Hemitoxin (HTx), a K^+^ channel blocker, was isolated from the venom of *Hemiscorpius lepturus*, which is classified in subfamily six of the α-KTx family of potassium channel scorpion toxins and has the highest amino acid sequence similarity with maurotoxin (MTX), extracted from the Tunisian scorpion *Scorpio maurus palmatus*. Additionally, MTX is also a K^+^ channel inhibitor with 34 (instead of 35) amino acid residues.

HTX reversibly inhibits type K_v_1.1, K_v_1.2, and K_v_1.3 channels. HTX has subtype-selective effects on K^+^ channels. It is 20 times less potent on K_v_1.2 channels, and 90 times more potent on K_v_1.3 channels, compared to the α-KTx6 family member MTX [118].

### 6.3. Ca^2+^ Release-Channel Specific Peptides (Calcins)

Voltage-gated calcium channels (Ca_v_) play a critical role in electrical signaling, converting depolarization of the cell membrane to an influx of calcium ions that initiates contraction, secretion, neurotransmission, and other intracellular regulatory events [92]. Voltage-gated calcium channels (Ca_v_) facilitate cellular calcium influx in response to membrane depolarization. They control hormone secretion, neurotransmitter release, propagation of cardiac action potential, muscle contraction, and gene expression in different cell types [119]. Similarly to the Na_v_ channels, the α1 subunit of Ca_v_ channels is organized in four homologous domains (I–IV), each containing six TM segments (S1–S6). The S1–S4 segments are involved in the voltage sensor, whereas S5–S6 constitutes the pore. Auxiliary subunits usually associate with α1, regulating channel expression and function. Ca_v_ channels are classified in the following groups in terms of electrophysiological and pharmacological properties and tissue distribution—L-type (Ca_v_1 subfamily: Ca_v_1.1-Ca_v_1.4); P/Q-, N-, and R-types (Ca_v_2.1, Ca_v_2.2 and Ca_v_2.3, respectively); and T-type (Ca_v_3 subfamily: Ca_v_3.1-Ca_v_3.3) [120].

The various subfamilies have been presented for classification of calcium channels as follows: Voltage-gated channels, voltage independent channels, and ligand-activated channels. Ligand-activated channels include the ryanodine receptors (RyRs), which are high-permeability Ca^2+^ channels of the sarcoplasmic reticulum in muscle and of the endoplasmic reticulum in other cells. In striated muscle fibers, contraction has been carried out via RyRs that releases Ca^2+^ rapidly. There are various isoforms of ryanodine-sensitive calcium channels including RyR_1_, RyR_2_, and RyR_3_. In mammals, RyR_1_ is expressed mainly in skeletal muscle and RyR_2_ is expressed mainly in cardiac muscle. RyR_3_ seems to be confined to the brain, smooth muscle, and epithelial cells. RyR_1_ and RyR_2_ are expressed in some of these tissues as well. The activity of surface-membrane Ca^2+^ channels, the dihydropyridine receptors (DHPRs), stimulates the activation of RyRs during the early part of the excitation–contraction linking cascade [121,122].

There is selective activity on ryanodine receptors (RyRs) by the venom of *Hadrurus gertschi* [123].

In *Odontobuthus doriae*, one calcium channel toxin was recognized and is referred to as ODCaTx_1_ (ID: KU365856) [124]. It shares 91% of its identity with the ryanodine receptor toxin isoform 2 isolated from *Hottentotta judaicus* [125]. Analysis and characterization of this peptide could produce remarkable biological and therapeutic research [124].

Hemicalcin is a new toxin that was extracted from the venom of *Hemiscorpius lepturus*, it represents 0.6% of the total protein content [126].

### 6.4. Cl^−^ Channels (CLCs)

CLC chloride channels contain one pore per subunit (a ‘double-barreled’ channel), and also provide clues about gating and permeation [127]. Cl^−^ channels are classifed into three groups: Ligand-gated Cl^−^ channels, the cystic fibrosis transmembrane conductance regulator (CFTR) channel, and the CLC channels [128]. The mammalian CIC family contains nine members, divided into three subgroups. It includes plasma membrane channels and Cl^−^/H^+^ antiporters that are thought to contribute to plasma membrane transport, lysosomal acidification, and the maintenance of the cell membrane potential [128,129,130].

Short scorpion toxin chloride channel inhibitors are short-chain neurotoxins (SCNs) that block small-conductance chloride channels. They are 30-40-residue long and contain four intramolecular disulphide bridges, which have been labeled as C1-C4, C2-C6, C3-C7, and C5-C8 [131,132,133].

According to Naderi Soorki et al. [124], one chloride channel-acting toxin, ODClTx1, was identified in *Odontobuthus doriae* venom (ID: KU-365857); it is composed of four intramolecular disulfide bridges and a putative conserved domain belonging to the toxin-5 superfamily. There are a variety of secreted short scorpion toxins in the superfamily. Such toxins are not associated with the toxin-2 superfamily (pfam00451) that affects potassium channels [124].

## 7. Antimicrobial Peptides

These days, antimicrobial components represent one of the important resources for modern medical care [134]. Natural antimicrobial peptides (AMPs) have been extracted from scorpion venom, are broadly expressed, and exert different effects on bacteria, viruses, fungi, and parasites [135]. Recently, 871 peptides/proteins have been discovered from 72 scorpion species [71]. Of these molecules, 638 peptides (73%) are from 47 scorpion species of the Buthidae family [71]. These consist both of disulfide-bridged peptides (DBPs), and of non-disulfide-bridged peptides (NDBPs) [55,136,137]. According to Almaaytah and Albalas [52], DBPs cause neurotoxic effects, and NDBPs reveal diverse structures and activities. There is generally more antimicrobial activity in NDBPs, which contain 13–56 amino acid residues; their amino acid sequences show structural diversity and multifunctional activity. So far, more than 40 peptides have been recognized and functionally categorized from scorpion venoms. Therapeutic and biological applications of NDBPs are related to their antibacterial, antifungal, antiviral, insecticidal, antimalarial, anticancer, cytolytic, anti-inflammatory, immune-modulatory, and bradykinin potentiating activities [52].

Globally, approximately 36.7 million people suffer HIV, but still there is no definite cure to eradicate HIV transmission [138]. The five antimicrobial peptides (AMPs) of scorpion venoms have been separated to evaluate potential anti-HIV effects. Three of them (mocoporin-M1, BmKn_2_, and Kn_2-7_) showed powerful anti-HIV activity [78].

Moreover, some distinctive characteristics of scorpion physiology are related to the mixture, and to individual effects. These properties are mostly found in the Buthidae family, whose venom is pharmacologically the most important and prominent compared to non-buthids. Hemocyanin is a protein for transporting oxygen in all scorpions and has the ability to bind with molecular oxygen in reverse. The protein has three parts with an enzymatic role: Pseudo-catalase, peroxidase, and superoxide-dismutase [139,140,141]. Additionally, the hemocyanins probably have an antimicrobial function from multiple oxidative enzymatic activities [142].

*Hemiscorpius lepturus* scorpion venom contains several components with significant anti-HIV activity, suggesting it as a potential source of novel therapeutic agents against HIV infection [143].

An antimicrobial toxin extracted from *Mesobuthus eupeus* venom glands was isolated that was encoded by a 213 bp cDNA fragment. The full-length sequence of the coding region was 210 bp and included an open reading frame of 70 amino acid residues with a predicted molecular mass of 7970.48 Da and theoretical pI of 9.10. The precursor (70 amino acid residues) includes a signal peptide of 23 amino acid residues and a mature peptide of 34 amino acid residues with no disulfide bridge. The resulted peptide of *Mesobuthus eupeus* has been named MeVAMP-2 (98%), MeVAMP-9 (60%). The other AMPs have been reported from *Mesobuthus martensii* (94%) and *Buthus occitanus israelis* (82%) [144].

There are six AMPs in venom of *Odontobuthus doriae*, called ODAMP1-6. Four of them (ID: KU212813, KU212814, KU212815, KU212816) have signal peptides with peptides containing 55, 51, 52, and 51 amino acid residues, respectively. There are no signal peptides in ODAMPs (ODAMP1, 6). ODAMP1 (ID: KU212812) has 78 amino acid residues and resembles the antimicrobial peptide androcin 18-1 from *Androctonus bicolor* scorpion (78% identity). ODAMP6 (ID: KU365855) is a short chain peptide with only 47 amino acid residues, which is similar to Tx65 with antimicrobial activity from *Buthus occitanus israelis* (100% identity), and which could be beneficial and applicable in drug and food industries [124].

## 8. Metalloproteinases

Matrix metalloproteinases (MMPs) affect cellular activities like growth and cell differentiation directly or indirectly [145,146,147]. In addition, MMP plays a role in cellular connection to the matrix using proteolysis of the adhesion places [148]. The enzymes can effect an increase and survival of tumor cells [149]. Metalloproteases of venom component play important role in hemorrhage [150]. Metalloproteinase sequences that have been identified in the transcriptome of *Odontobuthus doriae* include ODVP4; KU365871 [124] and those in *Hemiscorpius lepturus* include HLMP1; KX924496, HLMP2; KX924497, HLMP3; KX924498 [151,152,153].

## 9. Phospholipase A2 (PLA2)

Phospholipases (PLA) (types A_1_, A_2_, C, and D) are a type of enzyme with a high disulfide bridge content and a conserved histidine/aspartic catalytic dyad. They that act on phospholipids to produce different products, including lysophospholipids, diacylglycerols (DGs), free fatty acids (FFAs), choline phosphate, and phosphatidates [154,155]. The four main groups of superfamily Phospholipase A_2_ (PLA_2_) include secreted (sPLA_2_), cytosolic (cPLA_2_), calcium independent (iPLA_2_), and platelet activating factor acetyl hydrolase (PAF-AH) or lipoprotein PLA_2_ (LpPLA_2_). In vertebrates and invertebrates, the most common type of PLA is sPLA_2_, which is grouped into 15 types [156,157,158,159].

Secretory phospholipase A2 (sPLA2) is the most common type of phospholipase observed in animals including vertebrates and invertebrates. According to primary sequences alignments, disulfide bond patterna, and their biochemical properties, it is divided into 15 groups [157,159]. The low molecular weight of sPLA2 is13–15 kDa, with approximately six to eight disulfide bonds [154].

sPLA2 of scorpion venom are considered as group III and contained a long enzymatic chain and a short covalently fixed C-terminal chain generated after the release of five residues (penta-peptide) during the maturation processes [160]. HfPLA2 has been extracted from a scorpion named *Heterometrus fulvipes* [161], MtPLA2 from *Mesobuthus tamulus* [162], Imperatoxin (IpTxi) [163] and Phospholipin from *Pandinus imperator* [164], Phaiodactylipin from *Anuroctonus phaiodactylus* [165], Heteromtoxin (HmTx) from *Heterometrus laoticus* [166], Hemilipin from *Hemiscorpius lepturus* [167], and Sm-PLVG from *Scorpio maurus* [168]. *Hemiscorpius lepturus* has potent phospholipase D activities that have been related to the highly toxic (even lethal) necrosis activity of the venom [169].

In addition, a novel sPLA_2_ named hemilipin was recently isolated from dangerous scorpion of Iran, *Hemiscorpius lepturus* [167]. Edman degradation revealed its primary structure, and titration of fatty acids elucidated its enzymatic PLA_2_ activity on egg yolk phospholipids. Hemilipin widely affects angiogenesis in vitro and in vivo, whereas it doesn’t have any effect on apoptosis. Additionally, the study demonstrated that this new non-toxic sPLA_2_ could be used as an innovative tool to disrupt human angiogenesis at various points [167]. In a subsequent study, Jridi et al. [170] proposed a second sPLA_2_: Hemilipin_2_. This component has a robust calcium-dependent PLA_2_ activity and influences angiogenesis without any cytotoxic or apoptotic effects both in vitro and in vivo. However, there is a prominent capability in hemilipin_2_ to prevent blood vessel formation both in vitro and in vivo. The results suggest a beginning point to produce novel molecules that act as specific suppressors of human angiogenesis.

## 10. Protease and Serine Protease Inhibitors

Proteases are a kind of enzymes which are necessary to preserve homeostasis in cell. So far, 12 protease therapies have been proposed by the U.S. FDA (Food and Drug Administration) [171]. Based on Cao et al. [172], proteases control cellular events by growth factors, cytokines, chemokines, and cellular receptors, both through activation and inactivation leading to downstream intracellular signaling and gene regulation. Upregulation of proteolysis is related to different types of cancer and tumor metastasis, invasion, and growth [173].

Protease peptide inhibitors that occur in scorpion venoms have broad applications in medicine. Accordingly, proteases and protease inhibitors have important affects in pharmacology [171].

Protease inhibitors (PIs) are kinds of proteins or peptides which can be used to inhibit the catalytic activity of proteolytic enzymes [174]. SPIs have been found in scorpions and are classified in two groups—(1) Kunitz-type inhibitors, and (2) Ascaris-type inhibitors [174].

### 10.1. Kunitz-Type Inhibitors

Kunitz-type inhibitors are a group of serine protease inhibitors that are specified by a conserved spacing between their cysteine residues. There are one or more Kunitz domains in the inhibitor and these possess a typical disulfide bonding pattern [175,176]. Kunitz-type inhibitors are frequently found in arthropod venoms. In the scorpion and spider venoms, these peptides have dual functions [177].

### 10.2. Primary Sequence of Kunitz-Type Inhibitors

All the Kunitz-type protease inhibitors of scorpions have been recognized by the primary amino acid sequences of some inhibitor peptides that have been found in the NCBI protein data bank [174].

### 10.3. Ascaris-Type Inhibitors

Ascaris-type peptides usually possess a conserved structure with four short β-strands organized in two approximately vertical β-sheets and stabilized by five disulfide bridges: C1–C7, C2–C6, C3–C5, C4–C10, and C8–C9 [178].

### 10.4. Primary Sequence of Ascaris-Type Inhibitors

The sequences of all the Ascaris-type protease inhibitors of scorpions which have been found in NCBI protein data bank [174].

### 10.5. Functional Diversity of Protease Inhibitors

Some of Kunitz-type protease inhibitors of scorpions have been found to inhibit potassium channel K_V_1.3 [179]. Seven Kunitz-type protease inhibitors (LmKTT-1a, LmKTT-1b, LmKTT-1c, BmKTT-1, BmKTT-2, BmKTT-3, and Hg1) were tested on voltage-gated potassium channel subtype 1.3 (K_V_1.3 channel) and it was found that six of seven scorpion toxins, excepting rBmKTT-3, which had weak activity, inhibited ~50–80% of Kv1.3 channel currents at a concentration of 1 μM [174].

The serine proteinase inhibitor (serpin) superfamily participates in various necessary biological processes such as blood coagulation, complement activation, fibrinolysis, angiogenesis, inflammation, and tumor suppression. The members of this superfamily are expressed in a cell-specific manner [180]. Because of the abundance of Kunitz-type protease inhibitors in several organisms, they are the best-characterized family of serine protease inhibitors [181]. Two full-length coding sequences of *Hemiscorpius lepturus* transcriptome were coded as serine proteinase (KX932440 and KX932441 [152].

However, four putative serine protease inhibitors were discovered from the venom of three scorpion species including SjAPI (*Scorpiops jendeki Ascaris*-type protease inhibitor), SjAPI-2 (*Scorpiops jendeki Ascaris*-type protease inhibitor 2), CtAPI (*Chaerilus tricostatus Ascaris*-type protease inhibitor), and BmAPI (*Buthus martensii Ascaris*-type protease inhibitor) [182].

## 11. Scorpionism in Iran, a Major Public Health Problem

The risk of scorpion stings in rural areas is higher than in urban regions, and also more common in summer [183]. Much attention has been paid to the mortality rate of scorpion stings, whereas the incidence of scorpion stings is generally underestimated [8].

Scorpions are the most dangerous venomous animals for humans after snakes (venomous snakes) [184].

Climatic conditions, dryness, and heat are factors that increase the threat of scorpion stings [185]. Khuzestan is a province that is located in Southwestern Iran, along with the Persian Gulf region, with a hot and tropical climate. Scorpion stings are a main public health issue in the region especially for children and young adolescents [186]. In the Old World, Iran is acknowledged as one of the world’s hotspots for scorpionism [187].

**Figure 1:**
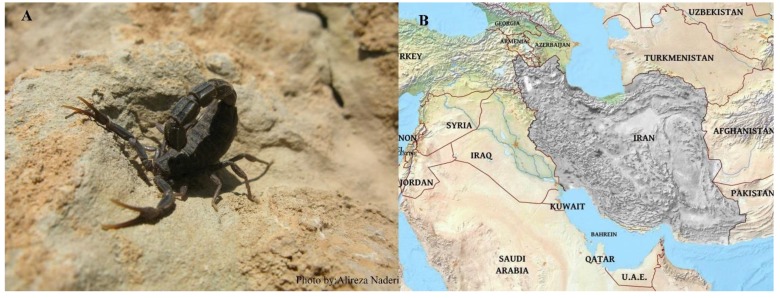
*Androctonus crassicauda* in its natural habitat (**A**) and its distribution map in gray (**B**).

**Figure 2:**
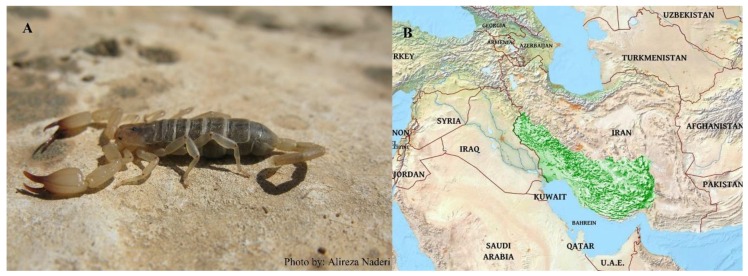
Image of *Hemiscorpius lepturus* in its natural habitat (**A**) and its distribution map in green (**B**).

Annually, more than 42,500 scorpion stings from 2001 to 2009 have been reported with about a 19.5% fatality rate. *Hemiscorpius lepturus*, *Androctonus crassicauda*, *Mesobuthus eupeus*, *Odontobuthus doriae*, *Hottentotta saulcyi*, *Hottentotta schach*, *Compsobuthus matthiesseni*, *Olivierus caucasicus*, *Orthochirus scrobiculosus*, and *Apistobuthus pterygocercus* are significant species in terms of medical and pharmacological relevance. Among Iranian scorpions, *Androctonus crassicauda* and *Hemiscorpius lepturus* have the highest risk of envenoming humans [188,189]. Distribution of *Androctonus crassicauda* (Figure 1) and *Hemiscorpius lepturus* (Figure 2) in Iran.

According to Shahbazzadeh et al. [190], 12,150 scorpion stings were reported from medical centers in six cities in the Khuzestan province in 2003. The prevalence of human scorpion stings is 3.1/1000 residents. By region, the highest prevalence is in Masjed-Soleiman (27.1%), followed by Ramhormoz (26.6%), Izeh (15.3%), Shush (12%), Baghmalek (11.7%), and Behbahan (7.3%). The most scorpion stings are inflicted by *Mesobuthus eupeus*, *Hottentotta saulcyi*, *Odontobuthus doriae*, and *Hemiscorpius lepturus*, responsible for 53.3%, whereas 17.4% were related to *Androctonus crassicauda* and *Hottentotta schach*, and 29.3% to other species. The maximum and minimum frequencies occur in June and February, respectively.

According to Mirshamsi et al. [48], specimens of *Mesobuthus eupeus* from Southwestern Iran belong to *Mesobuthus phillipsi*. Thus, all specimens of *Mesobuthus eupeus* in previous studies [144,191] should be considered *Mesobuthus phillipsi*. Based on Kovařík [23], all specimens of *Buthotus schach* or *Hottentotta schach* in prior research [100,101,188,189,190] should be considered *Hottentotta jayakari*, *Hottentotta khoozestanus*, and *Hottentotta zagrosensis*. Based on Lourenço [192], Mirshamsi et al. [38], and Navidpour and Lowe [193], there is no *Apistobuthus pterygocercus* in Iran and all specimens of *Apistobuthus pterygocercus* belong to *Apistobuthus susanae*, so all specimens of *Apistobuthus pterygocercus* in prior research [188,189] should be considered *Apistobuthus susanae*. The Razi Vaccine and Serum Research Institute, Hesarak, Karaj produces antivenoms of scorpions including six of the most dangerous scorpion species, including *Androctonus crassicauda, Hottentotta saulcyi, Hottentotta schach, Mesobuthus eupeus, Odontobuthus doriae,* and *Hemiscorpius lepturus* in Iran.

The Razi Vaccine and Serum Research Institute (Department of Venomous Animals and Antivenin Production) extracts venoms of *Hottentotta jayakari*, *Hottentotta khoozestanus*, *Hottentotta lorestanus*, and *Hottentotta zagrosensis*. These have previously been considered as *Hottentotta schach*, but occur in the southern provinces of Iran. Venoms of *Mesobuthus brutus*, *Mesobuthus caucasicus*, and, *Mesobuthus phillipsi* have been considered as *Mesobuthus eupeus*. According to Mirshamsi et al. [38,48] and Fet et al. [45], specimens of *Mesobuthus eupeus* should be named *Mesobuthus brutus*, *Mesobuthus caucasicus*, and *Mesobuthus phillipsi* based on geographical distribution. Also, According to Kovařík [23] and Navidpour et al. [33,35], specimens of *Hottentotta schach* should be named *Hottentotta jayakari jayakari*, *Hottentotta khoozestanus*, *Hottentotta lorestanus*, and *Hottentotta zagrosensis*. The Department of Venomous Animals and Antivenin Production, Razi Vaccine and Serum Research Institute should recognize the specimens carefully and then produce high quality scorpion antivenoms.

In conclusion, studying toxins of scorpion species from Iran is a way to present patterns and connection between species of scorpion and their venoms, which could be useful for understanding the molecular and functional diversities of scorpion venom sources, their evolutions, and probably the connection between scorpion species and their toxins.

## Supplementary Materials

The following are available online, Table S1: List of Scorpion species of Iran. Asterisk indicates endemic species, Table S2: Number and percentage of scorpions in Iran.
