# Supplementary material for: Venoms of Iranian Scorpions (Arachnida, Scorpiones) and Their Potential for Drug Discovery
Source: Molecules. 2019 Jul 23;24(14):2670. doi: 10.3390/molecules24142670 (PMC6680535; doi:10.3390/molecules24142670)
Supplement: Supplementary file 1 [file molecules-24-02670-s001.pdf]

Table S1. List of Scorpion species of Iran. Asterisk indicates endemic species.

Family	Genus	Species/Subspecies
Family Buthidae C. L. Koch, 1837	*Androctonus* Hemprich and Ehrenberg, 1828	*Androctonus baluchicus* (Pocock, 1900)
		Androctonus crassicauda (Olivier, 1807)
		Androctonus finitimus (Pocock, 1897)
		Androctonus robustus Kovařík & Ahmed, 2013
	Anomalobuthus Kraepelin, 1900	**Anomalobuthus talebii* Teruel, Kovařík, Navidpour and Fet, 2014
	Apistobuthus Finnegan, 1932	**Apistobuthus susanae* Lourenço, 1998
	Buthacus Birula, 1908	*Buthacus macrocentrus* (Ehrenberg, 1828)
	Compsobuthus Vachon, 1949	**Compsobuthus garyi* Lourenço and Vachon, 2001
		Compsobuthus jakesi Kovařík, 2003
		**Compsobuthus kaftani* Kovařík, 2003
		Compsobuthus matthiesseni (Birula, 1905)
		**Compsobuthus persicus* Navidpour, Soleglad, Fet and Kovařík, 2008
		**Compsobuthus petriolii* Vignoli, 2005
		**Compsobuthus plutenkoi* Kovařík, 2003
		Compsobuthus rugosulus (Pocock, 1900)
	Hottentotta Birula, 1908	*Hottentotta jayakari jayakari* (Pocock, 1895)
		**Hottentotta juliae* Kovařík, Yağmur and Fet, 2019
		**Hottentotta khoozestanus* Navidpour, Kovařík, Soleglad and Fet, 2008
		**Hottentotta lorestanus* Navidpour, Nayebzadeh, Soleglad, Fet, Kovařík and Kayedi, 2010
		**Hottentotta navidpouri* Kovařík, Yağmur and Moradi, 2018
		Hottentotta saulcyi (Simon, 1880)
		**Hottentotta schach* (Birula, 1905)
		**Hottentotta sistanensis* Kovařík, Yağmur and Moradi, 2018
		**Hottentotta zagrosensis* Kovařík, 1997
	Iranobuthus Kovařík, 1997	**Iranobuthus krali* Kovařík, 1997
	Kraepelinia Vachon, 1974	*Kraepelinia palpator* (Birula, 1903)
	Liobuthus Birula, 1898	*Liobuthus kessleri* Birula, 1898
	Mesobuthus Vachon, 1950	**Mesobuthus agnetis* (Werner, 1936)
		**Mesobuthus brutus* Fet, Kovařík, Gantenbein, Kaiser, Stewart and Graham, 2018
		Mesobuthus caucasicus (Nordmann, 1840)
		Mesobuthus eupeus afghanus (Pocock, 1889)
		Mesobuthus eupeus eupeus (C. L. Koch, 1839)
		**Mesobuthus eupeus iranus* (Birula, 1917)

		**Mesobuthus eupeus mesopotamicus* (Penther, 1912)
		Mesobuthus eupeus pachysoma (Birula, 1900)
		Mesobuthus eupeus persicus (Pocock, 1899)
		Mesobuthus eupeus philippovitschi (Birula, 1905)
		Mesobuthus eupeus thersites (C. L. Koch, 1839)
		Mesobuthus macmahoni (Pocock, 1900)
		Mesobuthus parthorum (Pocock, 1889)
		Mesobuthus phillipsi kirmanensis (Birula, 1900)
		Mesobuthus phillipsi phillipsi (Pocock, 1889)
		**Mesobuthus vesiculatus* (Pocock, 1899)
	Odontobuthus Vachon, 1950	*Odontobuthus bidentatus* Lourenço and Pézier, 2002
		**Odontobuthus doriae* (Thorell, 1876)
		Odontobuthus odonturus (Pocock, 1897)
		**Odontobuthus tavighiae* Navidpour, Soleglad, Fet and Kovařík, 2013
		**Odontobuthus tirgari* Mirshamsi, Azghadi, Navidpour, Aliabadian and Kovarik, 2013
	Orthochirus Karsch, 1891	**Orthochirus carinatus* Navidpour, Kovařík, Soleglad and Fet, 2019
		**Orthochirus farzanpayi* (Vachon and Farzanpay, 1987)
		Orthochirus fuscipes (Pocock, 1900)
		**Orthochirus gantenbeini* Kovařík, Yağmur, Fet and Hussen, 2019
		Orthochirus glabrifrons <u>(Kraepelin, 1903)</u>
		**Orthochirus gruberi* Kovařík and Fet, 2006
		**Orthochirus iranus* Kovařík, 2004
		Orthochirus mesopotamicus (Birula, 1918)
		**Orthochirus navidpouri* Kovařík, Yağmur, Fet and Hussen, 2019
		**Orthochirus scrobiculosus dentatus* (Birula, 1900)
		Orthochirus scrobiculosus melanurus Levy and Amitai, 1980
		Orthochirus scrobiculosus persa (Birula, 1900)
		**Orthochirus stockwelli* (Lourenço and Vachon, 1995)
		**Orthochirus varius* Kovařík, 2004
		**Orthochirus zagrosensis* Kovařík, 2004
	Polisius Fet, Capes and Sissom, 2001	**Polisius persicus* Fet, Capes and Sissom, 2001

	Razianus Farzanpay, 1987	**Razianus zarudnyi* (Birula, 1903)
	Sassanidotus Farzanpay, 1987	**Sassanidotus gracilis* (Birula, 1900)
		**Sassanidotus zarudnyi* (Birula, 1903)
	Vachoniolus Levy, Amitai and Shulov, 1973	**Vachoniolus iranus* Navidpour, Kovařík, Soleglad and Fet, 2008
Family Scorpionidae Latreille, 1802	*Scorpio* Linnaeus, 1758	*Scorpio maurus kruglovi* Birula, 1910
		Scorpio maurus townsendi (Pocock, 1900)
Family Hemiscorpiidae Pocock, 1893	*Hemiscorpius* Peters, 1861	**Hemiscorpius acanthocercus* Monod and Lourenço, 2005
		**Hemiscorpius enischnochela* Monod and Lourenço, 2005
		**Hemiscorpius gaillardi* (Vachon, 1974)
		**Hemiscorpius kashkayi* Karatas and Mouradi-Gharkheloo, 2013
		Hemiscorpius lepturus Peters, 1861
		**Hemiscorpius persicus* Birula, 1903
		**Hemiscorpius shahii* Kovarik, Navidpour and Soleglad, 2017
Family Diplocentridae Karsch, 1880	*Nebo* Simon, 1878	**Nebo henjamicus* Francke, 1980

Table S2. Number and percentage of scorpions in Iran.

Family	Number of genera	Percentage of genera	Number of species/subspecies	Percentage of species/subspecies
Buthidae	16	84.2	68	87.17
Hemiscorpiidae	1	1.8	7	8.97
Scorpionidae	1	1.8	2	2.56
Diplocentridae	1	1.8	1	1.28
